# Association between Oral Chinese Herbal Medicine and Recurrence and Metastasis in Patients with Stages II and III Colorectal Cancer: A Cohort Study in China

**DOI:** 10.1155/2022/8529395

**Published:** 2022-11-08

**Authors:** Mo Tang, Wei Zhang, Wenyu Qin, Chao Zou, Yunzi Yan, Bin He, Yun Xu, Ying Zhang, Jianping Liu, Hong Sun, Yufei Yang

**Affiliations:** ^1^Xiyuan Hospital, China Academy of Chinese Medical Sciences, Beijing 100091, China; ^2^Beijing Cancer Hospital, Beijing 100142, China; ^3^Beijing University of Chinese Medicine, Beijing 100029, China; ^4^Epidemiology Department of Beijing University of Chinese Medicine, Beijing 100029, China

## Abstract

**Background:**

To evaluate the associations between long-term oral Chinese herbal medicines (CHMs) and recurrence and metastasis (R&M) in patients with stage II and III colorectal cancer (CRC). Furthermore, we aimed to determine the correlation between different syndrome patterns and prognosis and summarized the regularities among CHMs prescriptions, providing reference for clinical practice.

**Methods:**

An ambispective cohort study was conducted. All CRC patients who sought evaluation and treatment at Xiyuan Hospital and Beijing Cancer Hospital from August 2014 to August 2016 were included. In this study, “whether patients voluntarily take CHMs” was taken as the exposure factor, and the exposure degree was “the duration of CHM use.” Stratification was performed according to the duration of TCM use to determine the relationship with R&M of CRC. The primary outcome was disease-free survival. Patients who had R&M of CRC after taking CHMs for ≥6 months were defined as “worst patients.” *R* software was used for statistical analysis. The Kaplan–Meier method and Cox regression analysis were used to determine the prognosis. IBM SPSS was used to model a priori association rules; drug use rules were analyzed on this basis.

**Results:**

A total of 186 patients with stage II and III CRC after radical resection were enrolled. All patients reached the study endpoint by August 2021. The difference in disease-free survival between the two groups was most significant when the cutoff value for CHMs was 18 months (*P* = 0.0012). Multivariate analysis showed that 18 CHMs were independent protective factors for R&M of CRC (*P* = 0.001, HR = 0.20, 95% CI = 0.08–0.53). The ratio of Pi (spleen) and Shen (kidney) deficiency in the worst cases was higher than patients without R&M (*P* = 0.018). Sijunzi and Liuwei Dihuang decoctions were the most frequently used prescriptions in the anti-R&M phase.

**Conclusion:**

CHMs complying with the “Jianpi Bushen” principle may attenuate the risk of R&M in patients with stage II and III CRC. Pi (spleen) and Shen (kidney) deficiency in patients receiving TCM intervention for the first time within 6 months of radical resection may be associated with a higher CRC R&M rate. Further research is warranted to validate these findings and elucidate underlying biological mechanisms.

## 1. Introduction

Colorectal cancer (CRC) is the third most common cancer and the second leading cause of cancer-related deaths [1]. Despite advances in treatment and implementation of screening and surveillance [2], CRC remains a major global health problem with high morbidity and mortality [3]. Patients with nonmetastatic CRC are advised to undergo standard radical surgery with or without adjuvant chemotherapy; however, recurrence and metastasis (R&M) occurs in 30%–50% of such patients receiving optimal treatment, and <20% of patients with advanced CRC survive 5 years after diagnosis [4, 5]. Furthermore, despite standard treatment, many CRC patients still have physical symptoms, such as fatigue, insomnia, and flatulence, as well as psychological distress [6], which negatively impact patients' well-being [7]. Thus, prolonging the disease-free survival (DFS) and improving the quality of life are worthy goals for patients with nonmetastatic CRC.

Traditional Chinese medicine (TCM), as a long-standing science and culture, plays an important role in complementary and alternative medicine therapies in China, where TCM is widely accepted by patients and covered by Chinese health insurance. Because of numerous properties, TCM has an increasingly prominent role in various diseases, including CRC [8]. A large cancer center conducted parallel surveys and reported that 83% of patients used TCM, with oral Chinese herbal medicines (CHMs) accounting for 55.8% of the total [9]. CHMs have gained increasing acceptance in the clinic for their proven efficacy in CRC treatment [10]. Our previous study demonstrated that CHMs are associated with a lower R&M rate [11]. We then conducted a multicenter prospective cohort study and reported that using CHMs for 1 year improved survival outcomes in patients with stage II and III CRC [12]. According to the TCM theory, cancer is caused by an imbalance between endogenous physical conditions of the body and exogenous pathogenic factors [12]. Shen (kidney) is the origin of congenital constitution, while Pi (spleen) is the root of acquired constitution. A healthy qi deficiency in TCM is mainly manifested by a decline in the autoimmune function caused by innate deficiency or acquired hypotrophy, which cannot monitor and annihilate tumor cells. As a result, there is spread and diffusion of circulating tumor cells and tumor dormant cells in the body, which eventually leads to tumor progression [13, 14]. Patients with nonmetastatic CRC can have tumors excised through conventional Western medicine practices, but the patients may have spleen deficiency and residual disease. Thus, several previous studies have opined that CRC patients can be treated with the “Jianpi Bushen” rule [15, 16].

TCM emphasizes individuality and places a high value on the holistic view. Syndrome differentiation refers to diagnosing an illness as a specific syndrome based on an analysis of the specific symptoms and physical signs collected through inspection, auscultation and olfaction, inquiry, and palpation, while treatment refers to defining the treatment approach in line with the syndrome differentiated. This view coincides with the principle of precision medicine in modern medicine. But it is difficult to determine a common prescription that is effective. We performed a two-center ambispective cohort study with a maximum follow-up of five years to provide clinical evidence to show the advantages of TCM “Jianpi Bushen” theory against R&M in patients with stage II and III CRC. In addition, we used the concept of “target population” in enrichment design [17]. Patients using CHMs for more than five years without R&M were defined as the TCM “target population” in this study. The core drug groups with a common therapeutic background were extracted using data mining techniques to explore the regularity of CHM prescriptions in the TCM “target population.” This was achieved to reveal the similarities and differences in CHMs for CRC and provide a basis for the rational use of CHMs in clinical practice.

## 2. Methods

### 2.1. Study Population

Patients in this ambispective cohort were admitted to the Xiyuan Hospital of China academy of Chinese medical sciences and Beijing Cancer Hospital, Beijing, China between August 2014 and August 2016. The inclusion criteria were the following: (1) patients between the ages of 18–80 years, with no gender restrictions; (2) the primary, and only, tumor site was CRC, and pathologically diagnosed as adenocarcinoma (pathological wax and sections could be obtained); (3) TNM stages were stage II and III; (4) patients who received standard treatments within 6 months after radical resection (R0 resection), according to the National Comprehensive Cancer Network (NCCN) guidelines [18]. The exclusion criteria were the following: (1) patients with intestinal obstruction, who cannot take decoction and need intravenous nutrition; (2) patients who are allergic to the known Chinese medicine ingredients used in the study; (3) pregnant and lactating women with mental illness; and (4) those who participated in other clinical studies.

### 2.2. Study Design

Based on the cumulative use of CHMs for 6 months, we divided patients into an integrated Chinese and Western medicine or Western medicine group [19]. When two consecutive CHM treatments were >6 weeks apart, CHMs were considered discontinued [20]. Patients using CHMs for >5 years without CRC R&M were defined as the TCM “target population.” When stratified according to CHM use, the “worst cases” were defined as patients who had R&M after taking TCM for ≥6 months. Patients without CRC R&M within 5 years were compared with the worst cases. The regularity of CHM prescriptions in the TCM “target population” was summarized. TCM syndrome pattern criteria were in accordance with the clinical research guidelines for TCM New Drugs [21]. There are four types of TCM syndrome patterns: Pi (spleen) Qi deficiency; Shen (kidney) Yin deficiency; Pi (spleen) and Shen (kidney) deficiency; and non-Pi (spleen) and Shen (kidney) deficiency.

### 2.3. Follow-Up Strategy and Outcomes

Patients received follow-up according to NCCN clinical guidelines, with clinical visits every three months for the first two years, every six months between years 3 and 5, and then yearly after year 5. We recorded TCM syndrome patterns and detailed prescription information of TCM. All physicians participating in the study received training in advance. Patients who occasionally miss clinical visits are followed up by telephone by trained researchers. If the telephone follow-up failed three times, it was defined as lost to follow-up. DFS, defined as the time from intervention to tumor R&M or death from any cause, was considered as the primary end point.

### 2.4. Statistical Analysis

**Figure 1:**
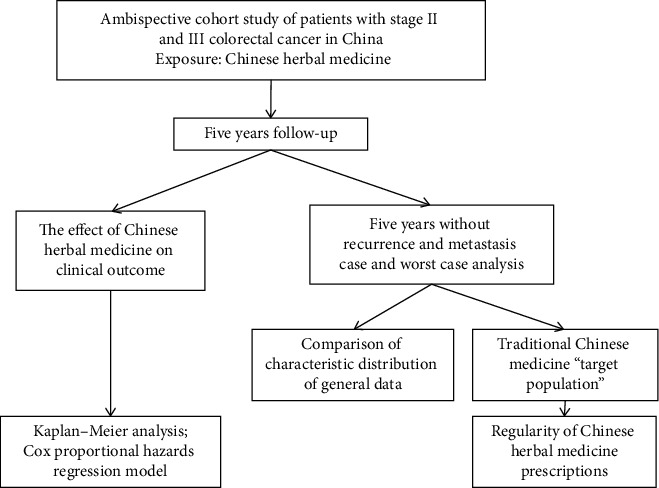
Study design.

Statistical data were described using the median for continuous variables and percentages (%) for categorical variables. Differences in the baseline characteristics were grouped by quartiles (*Q*) of cumulative months of CHM use (*Q*1, *Q*2, *Q*3, and *Q*4) and assessed using one-way analysis of variance, Kruskal–Wallis *H* test, *χ*^2^, or Fisher's tests to determine statistical differences between the groups. Kaplan–Meier survival analysis was performed using the surv-cutpoint function in the “survminer” *R* package, which determines the optimal cut point that yielded the minimum *P* value in the log-rank test between the high-andlow-risk subgroups [22]. Cox proportional hazard ratio (HR) with 95% confidence intervals (CIs) was used to determine the simultaneous impact of other variables potentially associated with each outcome. A *P* value <0.05 (two-sided) was considered statistically significant. Statistical analyses were analyzed using *R* software (version 3.6.2). Age was a continuous variable, which was converted to a categorical variable using X-tile (version 3.6.1, Yale University) [23]. Regularity of CHM prescriptions through data mining was analyzed by the “Apriori block,” which was provided by SPSS 18.0 software for cluster and network analyses [24]. The study design is illustrated in Figure 1.

## 3. Results

### 3.1. Baseline Characteristics

**Figure 2:**
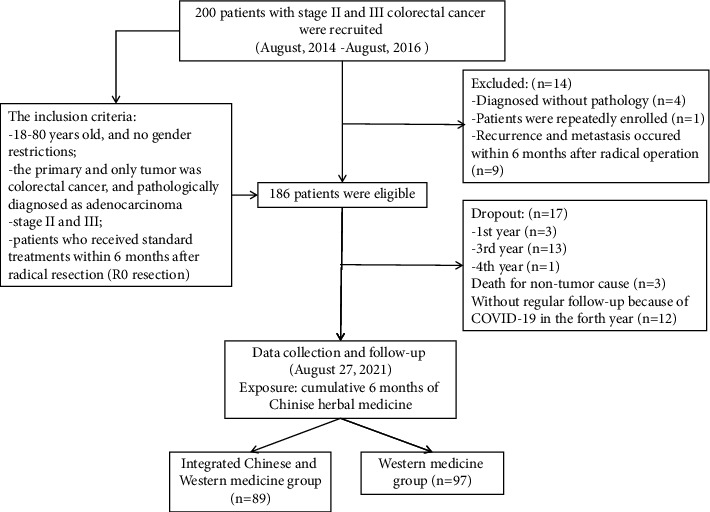
Flow chart of study participants.

A total of 200 patients from two hospitals with stage II and III CRC were enrolled in this cohort study between August 2014 and August 2016. Fourteen patients were further excluded from the analysis as they did not conform to the study protocol. A total of 186 patients were enrolled, including 89 integrated Chinese and Western medicine patients and 97 Western medicine patients. 17 patients dropped out during follow-up and 12 patients did not receive regular surveillance in the hospital during the last two years due to COVID-19. Three patients died due to nontumor causes. The flowchart of patient recruitment for this study is shown in Figure 2. The optimum cutoff score for age was determined to be 60 years. The baseline characteristics of participants based on quartiles of cumulative days of CHM use are summarized in Table 1.

### 3.2. Impact of CHM Treatment on Clinical Outcomes

**Figure 3:**
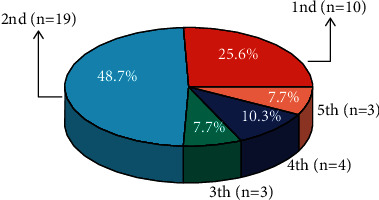
Pie chart of proportion of patients with CRC R&M within 5 years of surgery.

**Figure 4:**
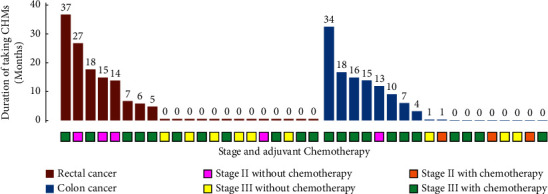
Disease data of 39 patients with CRC R&M and duration of using CHMs.

**Figure 5:**
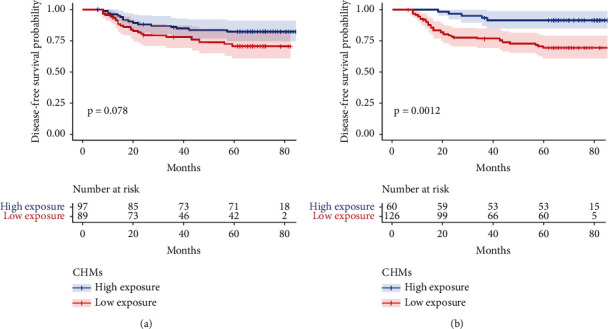
Kaplan–Meier curves of disease-free survival (DFS) in patients with stage II and III colorectal cancer stratified by Chinese herbal medicine (CHM) use during the follow-up period. The *x*-axis represents the time since surgery (months). (a) Exposure to CHMs is for 6 months; (b) cut-off point is 18 months of CHM use.

On August 27, 2021, we collected the final data. The median DFS follow-up is 65.0 (2.0–89.0) months. During the follow-up period, 39 of the 186 patients in this study experienced cancer R&M: the number of patients with R&M was 10 (25.6%), 19 (48.7%), 3 (7.7%), 4 (10.3%), and 3 (7.7%) per year from 1 to 5 years after radical resection, respectively (Figure 3). Among 39 patients with CRC R&M, there were 21 with rectal cancer and 18 with colon cancer. There were eight patients with stage II CRC and 31 patients with stage III CRC (Figure 4). Kaplan–Meier analysis demonstrated that the integrated Chinese and Western medicine group had a lower incidence of CRC R&M than the Western medicine group (*P*=0.078; Figure 5(a)). Using a cutoff point of 18 months and the use of CHMs determined by the “survminer” package, patients were divided into low and high exposure groups. Kaplan–Meier curves illustrated that patients with low exposure had a significantly worse DFS than those with ≥18 months CHMs (*P*=0.0012; Figure 5(b)).

### 3.3. Multivariate Analyses

**Figure 6:**
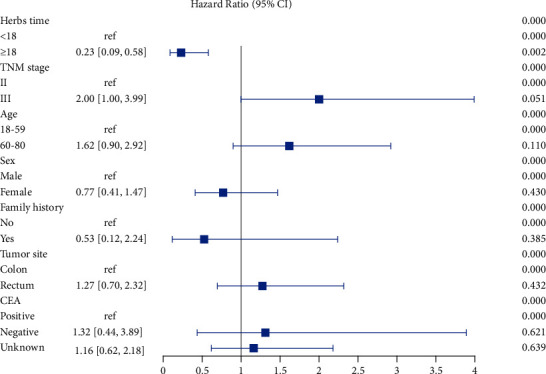
Forest plot for subgroup analyses. Multivariable hazard ratios (HRs) and 95% confidence intervals (CIs) for disease-free survival (DFS) across strata of various factors. Analyses used seven categories. ref: reference.

We examined the influence of CHMs on DFS across strata of other factors, including TNM stage, age, sex, family history of CRC, tumor site, and carcinoembryonic antigen (CEA). The use of CHMs for 18 months was the only factor associated with a significant improvement in DFS (HR, 0.23; 95% CI, 0.09–0.58; *P*=0.002). No other statistically significant interactions were detected. A forest plot of multivariable analyses in these subgroups is shown in Figure 6.

### 3.4. Comparative Analysis of Patients without CRC R&M within 5 Years of Surgery and the Worst Cases

A total of 113 patients did or did not receive CHM intervention, and no CRC R&M occurred within 5 years after radical surgery. CRC R&M occurred in 14 patients who received TCM intervention for ≥6 months (the worst case). Among the 113 patients, 65 (57.5%) were <60 years of age and 48 (42.5%) were >60 years of age. There were 47 cases in females (41.6%) and 66 cases in males (58.4%). The tumor was localized to the colon and rectum in 59 (52.2%) and 54 cases (48.8), respectively. There were 49 stage II (43.4%) and 64 stage III CRC patients (56.6%). Among the 14 cases, 6 (42.9%) were <60 years of age and 8 (57.1%) were ≥60 years of age. There were two female (14.3%) and 12 male patients (85.7%). Localization of the tumor to the colon and rectum were equally distributed in 7 patients. There were four patients with stage II (28.6%) and 10 patients with stage III CRC (71.4%). Among 113 patients, 75 (66.4%) received adjuvant chemotherapy and 38 (33.6%) did not receive adjuvant chemotherapy. Among the worst patients, 10 (71.4%) received adjuvant chemotherapy and 4 (28.6%) did not receive adjuvant chemotherapy. The proportion of Pi (spleen) and Shen (kidney) deficiency in the worst cases was higher than the patients without CRC R&M within 5 years of surgery (*P*=0.018). There were no differences in the distribution of other features, as shown in Table 2.

### 3.5. Data Mining of Prescription in the TCM “Target Population”

**Figure 7:**
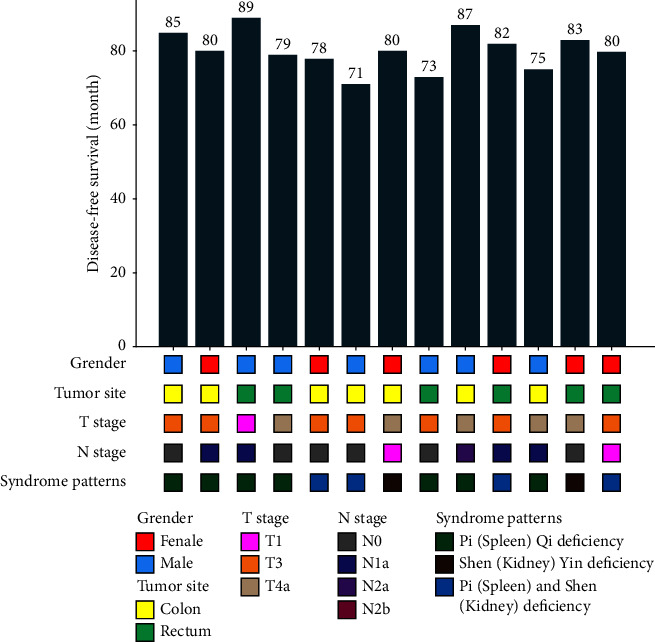
Data of patients who used CHMs for ≥5 years without recurrence and metastasis.

A total of 13 patients were in the TCM “target population,” namely, patients using CHMs for more than five years without R&M. The data of 13 patients were analyzed, including six women (46.2%), seven men (53.8%), seven patients (53.8%) with tumor localized to the colon, and six patients (46.2%) with tumor localized to the rectum. There were seven stage III CRC patients (53.8%) and six stage II CRC patients (46.2%). Among TCM syndromes, seven patients (53.8%) had Pi (spleen) qi deficiency, two patients (15.4%) had Shen (kidney) yin deficiency, and five patients (30.8%) had Pi (spleen) and Shen (kidney) deficiency, as shown in Figure 7.

**Figure 8:**
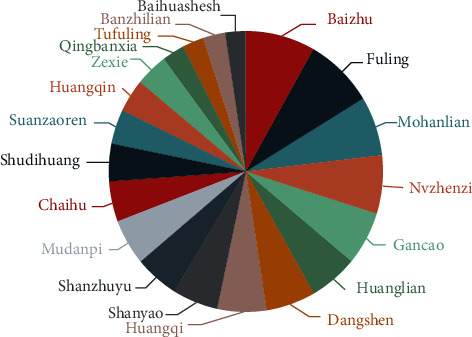
Proportion chart of top 20 CHMs.

**Figure 9:**
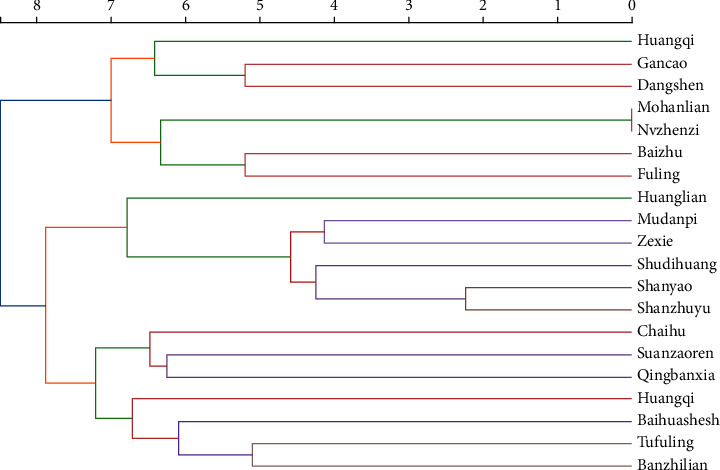
Network of Chinese herbal medicines in the “target population.”

A total of 406 prescriptions containing 215 herbs were collected. The frequency statistics showing the top 20 used herbs and their actions according to the Chinese Pharmacopoeia (2020 edition) are shown in Table 3 [25]. The analysis of association rules identified 20 high-frequency medicine pair rules and the complicated network for those are visualized in Figure 8. With the cluster analysis, we generated a dendrogram from which three clustering items were extracted with a relative distance of 7.5, among which two kinds of prescription contained more than five CHMs (Figure 9). The extracted prescriptions corresponded to three famous Chinese formulae: Sijunzi decoction, Liuwei Dihuang decoction, and Erzhi wan.

In Figure 9 Cluster analysis dendrogram of Chinese herbal medicines in the “target population”. The *Y*-axis indicates the serial number and herb name. There is a line of numbers at the top of the graph that indicates the relative distance for each class. This is the result of the proportional distance resetting for the relative distance of each class.

## 4. Discussion

In this ambispective cohort study, 186 patients with stage II and III CRC were followed up for five years. Furthermore, we found a positive relationship between CHMs and the risk of R&M in stage II and III CRC. We further observed that CHMs intervention for 18 months (as the cutoff value) was associated with the lowest risk of clinical outcome. Moreover, these associations were independent of other disease factors. In this study by comparing the TCM syndrome patterns of patients without CRC R&M within 5 years with the worst cases, it was found that the syndrome patterns of Pi (spleen) and Shen (kidney) deficiency were more common in the worst cases. Due to the limited number of patients, the exact relationship between TCM syndrome type and CRC R&M warrants further study by expanding the sample size. Our findings on the distribution of syndromes in CRC patients were similar to a study based on 760 CRC patients from two cancer centers in China [26].

This is the first study to summarize the prescription regularity of CHMs by patients to prevent CRC R&M, which is an important preference for TCM clinical practice. TCM theory holds that “The region where pathogenic factors invade must be deficient of Qi”. Pi (spleen) qi deficiency is a pathologic change characterized by qi deficiency combined with impaired transporting and transforming function of the spleen [27]. Patients' gastrointestinal function needs to be restored after radical surgery, and chemotherapy-induced nausea and vomiting are common side effects. Dizziness, weariness, indigestion, abdominal distension, lassitude, anorexia, diarrhea, and other Pi (spleen) qi-deficient disorders are common in CRC patients. Shen (kidney) yin deficiency is a pathologic change characterized by a deficiency of yin to nourish the kidney, leading to deficiency-fire or deficiency-heat, which is characterized by dizziness, forgetfulness, tinnitus, backache, and lack of libido, among other symptoms [27]. In addition, because cancer is a chronic wasting disease, CRC patients are predisposed to manifest Shen (kidney) yin deficiency syndrome. A total of 180 patients with curative rectal cancer operations were administered a questionnaire, most of whom experienced sexual dysfunction [28].

In TCM prescriptions, herbs are generally used in combination as “formulas”. Based on classical prescriptions, adding, subtracting, or changing medicine can achieve the therapeutic effect for different symptoms. In this study, we used data mining to reveal the underlying CHMs prescription regularity in patients with stage II and III CRC. We found that three classical and well-known prescriptions: Sijunzi decoction, Liuwei Dihuang decoction, and Erzhi wan were the most frequently used. The first two prescriptions were first recorded in the Song dynasty, while Erzhi wan was recorded in the Ming dynasty. Sijunzi decoction is the basic prescription for the treatment of Pi (Spleen) qi deficiency in TCM. It consists of Ginseng Radix et Rhizoma, Atractylodis macrocephalae rhizoma, Poria, and Glycyrrhizae Radix et Rhizoma. Ginseng Radix et Rhizoma is often replaced by Codonopsis radix in TCM clinical practice due to economic and source factors. Emerging research suggests that Sijunzi decoction is a possible treatment target for CRC and can potentially improve quality of life [29]. Liuwei Dihuang decoction is composed of six herbs: Rehmanniae radix praeparata, Dioscoreae rhizoma, Corni fructus, Moutan cortex, Poria and Alismatis rhizoma. Its use is to nourish yin and tonify the kidney. Several studies have proved that Liuwei Dihuang decoction may regulate some candidate molecular targets and participate in bioprocesses for the treatment of colon cancer [30]. A study based on network pharmacology investigation revealed that the mechanism of Sijunzi decoction and Liuwei Dihuang decoction refers to the CRC pathway [31]. The drug pair, Ecliptae Herba and Ligustri Lucidi fructus, which is also a traditional Chinese prescription called Erzhi wan, has been widely used to replenish the liver and kidney, nourish yin, and stop bleeding. The modern pharmacological study of Erzhi on CRC needs further exploration.

Several limitations to this study should also be considered. Our results may have been confounded by factors that were not captured in our clinical study, such as molecular genetics, physical activity, and dietary habits [32]. During the long-term CHM treatment, it is likely that patients were not fully compliant with prescriptions. Furthermore, due to the COVID-19 pandemic, several patients were unable to follow-up as scheduled, resulting in the clinical outcome being missed in evaluation. All the limitations mentioned above would have tended to overestimate the true associations between CHMs and R&M. Moreover, due to inadequate ambispective studies, patient information may be incomplete and data collection and follow-up may be biased.

## 5. Conclusions

The results of this ambispective cohort study suggested that CHMs may be associated with a lower risk of R&M in patients with stage II and III CRC. These findings are consistent with our previous studies. We further found that the effect on prolonging DFS was the greatest when the intervention using CHMs lasted for 18 months in patients. Our study attempted to further reveal the role of TCM in improving clinical outcomes by observing visceral pattern identification and concluding the regularity of CHMs prescriptions through data mining in patients undergoing five years of treatment for CRC prevention. It was demonstrated that “Jianpi Bushen” was the main method of treatment for patients with stage II and III CRC. Further research is required to confirm the generalizability of our findings.

## Figures and Tables

**Table 1:** Baseline characteristics of study participants according to quartiles of CHM use.

	*Q*1 (*n* = 81)	*Q*2 (*n* = 12)	*Q*3 (*n* = 47)	*Q*4 (*n* = 46)	Total (*n* = 186)	*P* value
Age at diagnosis, years						0.95
18–60	55 (42.3%)	9 (6.9%)	33 (25.4%)	33 (25.4%)	130 (69.9%)	
＞60	26 (46.4%)	3 (5.4%)	14 (25%)	13 (23.2%)	56 (30.1%)	
Gender						0.62
Female	32 (39.5%)	3 (25.0%)	18 (38.3%)	21 (45.7%)	74 (39.8%)	
Male	49 (60.5%)	9 (75.0%)	29 (61.7%)	25 (54.3%)	112 (60.2%)	
Tumor site						0.58
Colon	38 (46.9%)	7 (58.3%)	23 (48.9%)	27 (58.7%)	95 (51.1%)	
Rectum	43 (53.1%)	5 (41.7%)	24 (51.1%)	19 (41.3%)	91 (48.9%)	
Stage and adjuvant chemotherapy						0.003
Stage II without chemotherapy	3 (3.7%)	0 (0)	4 (8.5%)	7 (15.2%)	14 (7.5%)	
Stage II with chemotherapy	25 (30.9%)	2 (16.7%)	18 (38.3%)	14 (30.4%)	59 (31.7%)	
Stage III without chemotherapy	5 (6.2%)	2 (16.7%)	8 (17.0%)	12 (26.1%)	27 (14.5%)	
Stage III with chemotherapy	48 (59.3%)	8 (66.7%)	17 (36.2%)	13 (28.3%)	86 (46.3%)	
Syndrome patterns						0.03
Pi (spleen) qi deficiency	42 (51.9%)	8 (66.7%)	21 (44.7%)	25 (54.3%)	96 (51.6%)	
Shen (kidney) yin deficiency	12 (14.8%)	0 (0)	4 (8.5%)	4 (8.7%)	20 (10.8%)	
Pi (spleen) and Shen (kidney) deficiency	13 (16.0%)	4 (33.3%)	20 (42.6%)	14 (30.4%)	51 (27.4%)	
Non-pi (spleen) and Shen (kidney) deficiency	14 (17.3%)	0 (0)	2 (4.3%)	3 (6.5%)	19 (10.2%)	

Data are presented as the number (*n*) and percentage (%). *Q*: quartiles; CRC: colorectal cancer.

**Table 2:** Baseline data of patients without CRC R&M within 5 years of surgery compared with the worst patients.

	Patients without CRC R&M within 5 years of surgery (*n* = 113)	Worst patients (*n* = 14)	*P* value
Age at diagnosis, years			0.297
18–64	65 (57.5%)	6 (42.9%)	
＞64	48 (42.5%)	8 (57.1%)	
Gender			0.091
Female	47 (41.6%)	2 (14.3%)	
Male	66 (58.4%)	12 (85.7%)	
Tumor site			0.876
Colon	59 (52.2%)	7 (50.0%)	
Rectum	54 (47.8%)	7 (50.0%)	
TNM stage			0.440
Stage II	49 (43.4%)	4 (28.6%)	
Stage III	64 (56.6%)	10 (71.4%)	
Adjuvant chemotherapy			
Yes	75 (66.4%)	10 (71.4%)	0.938
No	38 (33.6%)	4 (28.6%)	
Syndrome patterns			
Pi (spleen) qi deficiency	59 (52.3%)	6 (42.9%)	0.509
Shen (kidney) yin deficiency	11 (9.7%)	0	0.609
Pi (spleen) and Shen (kidney) deficiency	30 (26.5%)	8 (57.1%)	0.018*∗*
Non-Pi (spleen) and Shen (kidney) deficiency	13 (11.5%)	0	0.358

**Table 3:** Top 20 CHMs used in the “target population.”

Mandarin Chinese names	Generic names	Functions	Frequencies
Fuling	Poria	To promote urination, to drain dampness, fortify the spleen, and calm the heart	79
Baizhu	Atractylodis macrocephalae rhizoma	To fortify the spleen, replenish qi, dry dampness, promote urination, stop sweating, and prevent miscarriage	79
Mohanlian	Ecliptae herba	To nourish the liver and kidney, cool the blood, and stanch bleeding	64
Nvzhenzi	Ligustri Lucidi fructus	To nourish the liver and kidney, improve vision, and blacken hairs	64
Gancao	Radix glycyrrhizae praeparata	To clear heat, drain dampness, and remove toxins	58
Huanglian	Coptidis rhizoma	To clear heat, drain dampness, purge fire, and remove toxin	56
Dangshen	Codonopsis radix	To fortify the spleen, replenish the lungs, nourish blood, and engender fluids	55
Huangqi	Astragali radix	To tonify qi, upraise yang, secure the exterior, stop sweating, promote urination, alleviate edema, engender fluids, nourish blood, move stagnation, relieve impediment, expel toxins and pus, promote wound healing, and promote tissue regeneration.	55
Shanyao	Dioscoreae rhizoma	To tonify spleen and stomach, engender fluids, nourish the lungs, tonify the kidneys, and astringe essence	52
Shanzhuyu	Corni fructus	To tonify and nourish the kidneys and liver, and astringe and prevent collapse	51
Mudanpi	Moutan cortex	To clear heat and cool the blood, activate the blood, and resolve stasis	49
Chaihu	Bupleuri radix	To disperse and reduce fever, soothe the liver, resolve depression, and upraise yang qi	45
Shudihuang	Rehmanniae radix praeparata	To nourish blood, replenish yin, and replenish the essence and the marrow	42
Suanzaoren	Ziziphi spinosae semen	To nourish the heart, tonify the liver, calm the heart, tranquilize the mind, relieve sweating, and engender fluid	39
Huangqin	Scutellariae radix	To clear heat, dry dampness, purge fire, remove toxins, stop bleeding, and prevent miscarriage	37
Zexie	Alismatis rhizoma	To promote urination, drain dampness, discharge heat, resolve turbidity, and lower lipids	36
Qingbanxia	Pinelliae rhizoma paeparatum cum alumine	To dry dampness and resolve phlegm	26
Tufuling	Smilacis glabrae rhizoma	To remove toxins, remove dampness, and relieve and facilitate joints	24
Banzhilian	*Scutellariae barbatae* herba	To clear heat, remove toxins, resolve stasis, and promote urination	23
Baihuasheshecao	*Hedyotis diffusa*	To clear heat, remove toxins, eliminate carbuncles, and drain dampness	23

## Data Availability

The datasets analyzed during the current study are available from the corresponding authors on reasonable request.

## Abbreviations

CHMs: Chinese herbal medicines
R&M: Recurrence and metastasis
CRC: Colorectal cancer
TCM: Traditional Chinese medicine
DFS: Disease-free survival
HR: Hazard ratio
CIs: Confidence intervals
CEA: Carcinoembryonic antigen.
